# A dimensional investigation of error-related negativity (ERN) and self-reported psychiatric symptoms

**DOI:** 10.1016/j.ijpsycho.2020.09.019

**Published:** 2020-10-17

**Authors:** T.X.F. Seow, E. Benoit, C. Dempsey, M. Jennings, A. Maxwell, M. McDonough, C.M. Gillan

**Affiliations:** a School of Psychology, Trinity College Dublin, Dublin, Ireland; b Trinity College Institute of Neuroscience, Trinity College Dublin, Dublin, Ireland; c St. Patrick ’s University Hospital, Dublin, Ireland; d Global Brain Health Institute, Trinity College Dublin, Dublin, Ireland

**Keywords:** Error monitoring, Error-related negativity, Transdiagnostic psychiatry

## Abstract

Alterations in error processing are implicated in a range of DSM-defined psychiatric disorders. For instance, obsessive-compulsive disorder (OCD) and generalised anxiety disorder show enhanced electrophysiological responses to errors—i.e. error-related negativity (ERN)—while others like schizophrenia have an attenuated ERN. However, as diagnostic categories in psychiatry are heterogeneous and also highly intercorrelated, the precise mapping of ERN enhancements/impairments is unclear. To address this, we recorded electroencephalograms (EEG) from 196 participants who performed the Flanker task and collected scores on 9 questionnaires assessing psychiatric symptoms to test if a dimensional framework could reveal specific transdiagnostic clinical manifestations of error processing dysfunctions. Contrary to our hypothesis, we found non-significant associations between ERN amplitude and symptom severity of OCD, trait anxiety, depression, social anxiety, impulsivity, eating disorders, alcohol addiction, schizotypy and apathy. A transdiagnostic approach did nothing to improve signal; there were non-significant associations between all three transdiagnostic dimensions (anxious-depression, compulsive behaviour and intrusive thought, and social withdrawal) and ERN magnitude. In these same individuals, we replicated a previously published transdiagnostic association between goal-directed learning and compulsive behaviour and intrusive thought. Possible explanations discussed are (i) that associations between the ERN and psychopathology might be smaller than previously assumed, (ii) that these associations might depend on a greater level of symptom severity than other transdiagnostic cognitive biomarkers, or (iii) that task parameters, such as the ratio of compatible to incompatible trials, might be crucial for ensuring the sensitivity of the ERN to clinical phenomena.

## 1 Introduction

Errors are a critically important information source. They allow us to monitor and continually adapt performance to changes in the environment, to improve skills slowly and incrementally, and to avoid large mistakes by having smaller ones attended to. Without this capacity, we might find ourselves repeating unproductive or damaging behaviours. Conversely, a hypersensitive error detection system might keep us from trying new things, from getting out of our comfort zone and experiencing the learning that comes from failure. Since the early nineties, the mainstay of error monitoring research has been the unique neural response to the commission of errors—the error-related negativity (ERN), a negative deflection of the event-related potential that peaks approximately 50–100 ms after an error response (Falkenstein et al., 1991; Gehring et al., 1993). Fundamentally, the ERN represents a well-validated and reliable neurophysiological index of error processing (Holroyd and Coles, 2002) with the anterior cingulate cortex posited to be its neural generator (Debener, 2005; Grützmann et al., 2016; Miltner et al., 2003).

Impairments in error monitoring are phenomenologically characteristic of a range of psychiatric disorders (Ullsperger, 2006), and this has been supported by the frequent observation of alterations in the ERN in patient groups (Gillan et al., 2017; Weinberg et al., 2015a). For example, studies have observed diminished ERNs in schizophrenia (Bates et al., 2002; Foti et al., 2012; Minzenberg et al., 2014; Morris et al., 2006; Simmonite et al., 2012), bipolar disorder (Minzenberg et al., 2014; Morsel et al., 2014) and substance use disorder (Franken et al., 2007; Sokhadze et al., 2008), while enhanced ERN amplitudes are consistently seen in obsessive-compulsive disorder (OCD) (Carrasco et al., 2013b; Endrass et al., 2014, 2008; Endrass and Ullsperger, 2014; Klawohn et al., 2014), social anxiety disorder (Endrass et al., 2014) and generalised anxiety disorder (Carrasco et al., 2013b; Weinberg et al., 2015b, 2012a, 2010). Though the precise functional role of the ERN is still highly debated (Alexander and Brown, 2011; Coles et al., 2001; Holroyd et al., 2005; Vidal et al., 2000; Yeung et al., 2004), there are several interpretations of the various ERN abnormalities observed in psychopathology. For diminished ERNs associated with bipolar disorder and schizophrenia, the phenomenon is hypothesised to reflect internal response monitoring deficits posited to underlie the generation of positive schizophrenia symptoms (Frith and Done, 1988; McGrath, 1991). As for disorders with enhanced ERN amplitudes (i.e. OCD, social anxiety and generalised anxiety), one commonality amongst these disorders is that they are characterised by high levels of anxiety. Here, the enhanced ERN is thought to reflect an increased sensitivity to errors (Hajcak, 2012; Weinberg et al., 2012b) which may be experienced as highly distressing (Dreisbach and Fischer, 2012; Hajcak and Foti, 2008; Spunt et al., 2012) in anxiety. This is supported by a body of evidence showing exaggerated physiological changes associated with anxiety (e.g. enhanced startle reflex (Hajcak and Foti, 2008; Riesel et al., 2013), heart rate deceleration (Hajcak et al., 2004, 2003a) and skin conductance changes (Hajcak et al., 2004, 2003a)) are linked to larger ERNs. Given that ERN amplitude shifts are so pervasive in psychiatry, it has been suggested and recognised by the Research Domain Criteria Initiative (RDoC) (Insel et al., 2010) that these reflections of altered error processing may be a transdiagnostic phenomenon (Gillan et al., 2017; Meyer and Klein, 2018; Weinberg et al., 2015a) that holds potential as a biomarker of mental health.

In recent years, meta-analyses have proposed phenotypes beyond Diagnostic and Statistical Manual of Mental Disorders (DSM) categories that may underlie alterations in ERN amplitude and explain their ubiquity across psychiatric groups. Particularly for the enhanced ERN, anxious apprehension (Moser et al., 2013) or uncertainty (Cavanagh and Shackman, 2015) are key candidates, supported by studies in non-clinical samples demonstrating that increased levels of worry (Hajcak et al., 2003b; Moser et al., 2012; Zambrano-Vazquez and Allen, 2014) and threat sensitivity (Weinberg et al., 2016) are associated with larger ERNs. That said, group-level effects in OCD patients tend to be more robust than in generalised anxiety disorder (Endrass and Ullsperger, 2014; Santesso et al., 2006), with a recent meta-analysis positing a higher effect size for OCD than anxiety (Pasion and Barbosa, 2019). As only a few studies have attempted to disentangle (and control for) intercorrelated symptoms within individuals in the same sample, and even fewer have done this in a sample of sufficient size, it remains to be seen if enhancements in the ERN confer risk for anxiety, compulsive symptoms or both.

To test this, we used a dimensional approach whereby we measured co-occurring symptoms of a range of disorders within the same individuals and tested for associations with the ERN in their original form, as well as after they had been reduced to three dimensions—anxious-depression, compulsive behaviour and intrusive thought (hereafter ‘compulsivity’) and social withdrawal (Gillan et al., 2016). Using this method, we previously showed that a transdiagnostic compulsive dimension maps onto deficits in goal-directed control better than OCD symptoms (Gillan et al., 2016); a finding that has since been replicated (Patzelt et al., 2019). We also showed that this method can reveal associations that are hidden by categorical disorder groupings. For example, anxious-depression is linked to reduced confidence, while individuals high on the spectrum of compulsivity have elevated confidence (Rouault et al., 2018; Seow and Gillan, 2020). This finding might explain why group level effects in OCD (where patients have high levels of both compulsivity and anxious-depression) have not revealed confidence abnormalities (Hauser et al., 2017; Vaghi et al., 2017). As such, the transdiagnostic method may be able to specify whether enhanced ERN amplitude shifts are truly related to anxious or compulsive symptomology. An additional advantage of using these previously defined transdiagnostic dimensions to disambiguate ERN relationships, as opposed to fitting new definitions of psychiatric phenotypes to our data here, is that it offers a clear extension to the several other cognitive phenomena (i.e. goal-directed control and metacognition) related to these dimensions. Generalising ERN effects to known cognitive mechanisms would foster better understanding of ERN amplitude shifts in psychopathology.

Following this methodology, we characterised participants in terms of a broad range of psychopathology (9 questionnaires in total) that have almost all been linked to the ERN in prior work; alcohol addiction, apathy, depression, eating disorders, impulsivity, OCD, schizotypy, social anxiety and trait anxiety. We hypothesised that an enhanced ERN would be associated with OCD, social anxiety and trait anxiety, but that this would be explained by a psychiatric dimension encapsulating high levels of anxiety, i.e. anxious-depression. While we expected OCD symptom severity to correlate with the ERN, we anticipated that the compulsivity dimension would not show an association as prior work has shown diminished ERN in addiction and schizophrenia (see review (Gillan et al., 2017)), both of which are strong contributors to the compulsivity dimension.

We related ERN amplitude to self-report psychiatric symptoms from 196 participants who completed the arrow-version of the Eriksen Flanker task (Eriksen and Eriksen, 1974). Contrary to our hypothesis, we found that none of the psychiatric symptoms nor the transdiagnostic dimensions were significantly associated to alterations in ERN amplitude. To contextualise the absence of ERN affects in the present sample (i.e. effect size), we report results from an additional cognitive task relating goal-directed learning (Daw et al., 2011) to dimensional phenotypes. Here, we did find evidence for an association; replicating prior work (Gillan et al., 2016) showing that goal-directed learning was related to the compulsive behaviour and intrusive thought dimension.

## 2 Materials and methods

### 2.1 Power estimation

An appropriate sample size was determined based on a previous study that reported an association of OCI-R scores and enhanced ERN amplitude that approached significance (*r* = 0.32, *p* = 0.06) (Gründler et al., 2009), an effect size suggesting that N = 155 participants were required to achieve 90% power at 0.005 significance.

### 2.2 Participants

The majority of participants were recruited from the general public through university channels via flyers and online advertisements, and a small number were patients from St. Patrick’s University hospital’s mental health service. We included these patients to ensure sufficient sampling of self-report mental health symptoms at the more severe end of the spectrum. They were all ≥18 years (with an age limit of 65 years) and had no personal/familial history of epilepsy, no personal history of neurological illness/head trauma nor personal history of unexplained fainting. After reading the study information and consent online, participants provided informed consent by clicking the ‘I give my consent’ button. They also gave written consent before the in-laboratory EEG session. They were paid €20 Euro (€10/h) upon completion of the study. We collected data from N = 234 participants; N = 8 were patients starting group treatment for anxiety from a local clinic and the rest N = 226 were from the general public. Of the total sample, 138 were female (58.97%) with ages ranging from 18 to 65 (mean = 31.42, standard deviation (SD) = 11.48) years. All study procedures were approved by Trinity College Dublin, School of Psychology Research Ethics Committee and St. Patrick’s University Hospital’s Mental Health Services Research Ethics Committee.

### 2.3 Procedure

Before arriving to the lab for testing, participants navigated a webpage to give informed consent, provide basic demographic data (age, gender), list any medications they were currently taking for *a mental health issue* (if so, to indicate the name, dosage and duration) and complete a set of 9 self-report psychiatric questionnaires. For a subset of the participants (N = 110, 47%), they completed a short psychiatric interview in-person on the day of testing (Mini International Neuropsychiatric Interview; M.I.N.I.) (Sheehan et al., 1998). During the experimental EEG session, participants completed two tasks: the modified arrow-version of the Eriksen flanker task (Eriksen and Eriksen, 1974) and the two-step reinforcement learning task (Daw et al., 2011). The latter task was analysed and published separately (Seow et al., 2020) and so the methods are not described in detail. However, we report one basic behavioural result from this task to contextualise our ERN results. Once participants completed both tasks, they completed a short IQ evaluation before being debriefed and compensated for their time.

### 2.4 Exclusion criteria

Several exclusion criteria were applied to ensure data quality. Participants were excluded if they failed any of the following on a rolling basis. (i) Participants whose EEG data were corrupted (N = 2) or incomplete (i.e. recording was prematurely cut) were excluded (N = 4). (ii) Participants whose error response-locked epochs over four electrode sites examined failed a threshold criterion of ±50 μV for 95% of epochs (see Response-locked ERPs) were excluded (N = 5). (ii) Participants who missed >20% of trials (n > 96) of the flanker task were excluded (N = 11). (iii) Participants who scored *<*55% accuracy were excluded (N = 9). (iv) Participants who incorrectly responded to a “catch” question within the questionnaires: “If you are paying attention to these questions, please select ‘A little’ as your answer” were excluded (N = 7). Combining all exclusion criteria, 38 participants (16.24%) were excluded. 196 participants were left for analysis (115 females (58.67%)), between 18 and 65 ages (mean = 30.82, SD = 11.53 years).

### 2.5 Disorder prevalence (M.I.N.I.)

After exclusion, 87 participants (44.39%) completed the M.I.N.I., which was introduced part-way through the study. Of these participants, 38 (43.68%) presently met the criteria for one or more disorder. Broken down by recruitment arm, 8 (100%) from the clinical arm met criteria, while 30 (37.97%) from university channels met criteria. This rate is close to published reports on the prevalence of mental health disorders in college student samples (Auerbach et al., 2018; Evans et al., 2018). Of the total sample, 31 (15.82%) were currently medicated for a mental health issue. Broken down by recruitment arm, all individuals recruited from the clinical setting were medicated, while 23 (12.23%) of those recruited through normal channels were medicated. Further diagnostic information of the sample is summarised in Supplemental Table S4.

### 2.6 Flanker task

Participants completed an arrow-version of the Eriksen Flanker task (Eriksen and Eriksen, 1974). Each trial consisted of either congruent (*<<* **<** *<<* or *>>* **>** *>>*) or incongruent (*<<* **>** *<<* or *>>* **<** *>>*) arrow stimuli presented in white on a grey background of a 32 × 24 cm computer monitor. Participants were instructed to respond as quickly and accurately as possible. Flanker stimulus were presented for 200 ms and they had 1050 ms to respond by pressing one of two keyboard keys in order to identify the direction of the central arrow. Responses were indicated using the left (‘Q’) and right (‘P’) keys. There were a total of 480 trials split into two blocks, each with 240 stimuli (80 congruent, 160 incongruent) presented. At the end of the first block, if participants had >25% missed trials or had accuracy >90%, they were told to ‘Please try to respond faster!’ for the second block. If their accuracy was *<*75%, they were told ‘Please try to respond more accurately!’. Otherwise they were told ‘Great job!’. Participants completed 30 practice trials (10 congruent; 20 incongruent) of a slower version of the task prior to the beginning of the experimental task (stimulus presentation: 400 ms, response time: 1000 ms).

### 2.7 Behavioural data pre-processing

Missed trials were excluded from analysis. A total of 2275 trials (2.42%) were removed (per participant mean = 11.61 trials).

### 2.8 EEG recording & pre-processing

Scalp voltage was measured using 128 electrodes in a stretch-lycra cap (BioSemi, The Netherlands). EEG signals were sampled at 512 Hz. EEG data were processed offline using EEGLab (Delorme and Makeig, 2004) version 14.1.2 in MATLAB R2018a (The MathWorks, Natick, MA). Data were downsampled to 250 Hz and high-pass filtered with a windowed sinc FIR filter at a pass-band edge at 1 Hz. Line noise was removed with CleanLine (Mullen, 2012) at frequencies 50, 100, 150, 200 and 250 Hz. Data were further pre-processed with Clean Rawdata plugin: bad channels were rejected with a criterion of 80% minimum channel correlation and continuous data were corrected using Artifact Subspace Reconstruction (ASR) (Mullen et al., 2013), with correction parameters set at 10 SD for burst criterion and 25% of contaminated channels for time window criterion. All removed channels were interpolated, and the data were re-referenced to the average. To reject ocular and other non-EEG artefacts, we ran ICA with *runica* (PCA option on) on unsegmented EEG data and rejected components automatically with Multiple Artifact Rejection Algorithm (MARA) (Winkler et al., 2011) at a threshold of >40% artifact probability.

### 2.9 Response-locked ERPs

To quantify ERN amplitudes, data were epoched response-locked from −400 ms to 500 ms and baseline adjusted using a −400 ms to −200 ms pre-response window on error trials. Epochs were rejected with a threshold criterion of ±50 μV before being averaged within-in participant. A total of 36 epochs (0.35%) were removed (per participant mean = 0.18 epochs). The minimum number of epochs for any participant was n = 9, which was above the recommended n = 6 for a reliable ERN (Olvet and Hajcak, 2009). We then used the adaptive mean method to estimate amplitude as it minimizes bias induced by individual-subject latency variability (Clayson et al., 2013). We searched for the largest negative peak within a window of − 20 ms to 120 ms post-response and took the mean amplitude ±40 ms of the negative peak’s latency. Correct-related negativity (CRN) amplitudes were measured with the same approach as the ERN, but on correct response trials. For the results reported in the main text, ERN and CRN activity were measured over the C23 (FCz) electrode. We also reported results from ERN activity at C23 obtained with other measurement methods (non-adaptive mean, minimum amplitude, trough to peak) and when controlled for CRN variation (ERN – CRN (ΔERN), residualised scores of ERN predicted by CRN (ERN_resid_)), as well as from over other electrodes (C22, C24 and D2, mean over 4 mid-frontal electrodes), in the Supplement.

### 2.10 Reliability measures

Internal consistency (split-half reliability) was calculated for the ERN and CRN. Data were split into two subsets (even versus odd trials/ epochs), correlated and adjusted with Spearman-Brown prediction formula.

### 2.11 Self-report psychiatric questionnaires & IQ

Participants completed self-report questionnaires assessing: *alcohol addiction* using the Alcohol Use Disorder Identification Test (AUDIT) (Saunders et al., 1993), *apathy* using the Apathy Evaluation Scale (AES) (Marin et al., 1991), *depression* using the Self-Rating Depression Scale (SDS) (Zung, 1965), *eating disorders* using the Eating Attitudes Test (EAT-26) (Garner et al., 1982), *impulsivity* using the Barratt Impulsivity Scale (BIS-11) (Patton et al., 1995), *obsessive-compulsive disorder* (OCD) using the Obsessive-Compulsive Inventory - Revised (OCI-R) (Foa et al., 2002), *schizotypy* scores using the Short Scales for Measuring Schizotypy (SSMS) (Mason et al., 2005), *social anxiety* using the Liebowitz Social Anxiety Scale (LSAS) (Liebowitz, 1987) and *trait anxiety* using the trait portion of the State-Trait Anxiety Inventory (STAI) (Spielberger et al., 1983). These self-report assessments were fully randomized within the psychiatric assessment component of the procedure and were chosen specifically to enable transdiagnostic analysis with psychiatric dimensions described in prior work (Gillan et al., 2016; Rouault et al., 2018). A proxy for IQ was also collected using the International Cognitive Ability Resource (I-CAR) (Condon and Revelle, 2014) sample test which includes 4 item types of three-dimensional rotation, letter and number series, matrix reasoning and verbal reasoning (16 items total). Correlations across questionnaires ranged highly (*r* = −0.05 to 0.75). Internal consistency for all questionnaires were high (Cronbach’s alpha > 0.81). Further details of correlations and reliability measures of questionnaires are in Supplemental Table S1.

### 2.12 Transdiagnostic factors (dimensions)

The current sample size was too small for de novo factor analysis (MacCallum et al., 1999). As such, raw scores of the 209 individual items from the 9 questionnaires were transformed into dimension scores (anxious-depression, compulsive behaviour and intrusive thought (‘compulsivity’), and social withdrawal) based on weights derived from a larger previous study (Gillan et al., 2016) (N = 1413). These dimensions are not orthogonal and correlate moderately (*r* = 0.33 to 0.39). See Supplemental Table S2.

### 2.13 Linear regressions

Regression analyses were conducted using linear models written in R, version 3.6.0 via RStudio version 1.2.1335 (http://cran.us.r-project.org) with the *lm()* function. We investigated if psychiatric questionnaire scores were related to ERN amplitude shifts by taking the total score for each questionnaire (*QuestionnaireScore*; z-scored) as a fixed effect predictor. Separate regressions were performed for each individual symptom due to high correlations across the different psychiatric questionnaires. The model was specified as: ERN ~ *QuestionnaireScore*. For the transdiagnostic analysis, we included all three dimensions in the same model, as correlation across variables was reduced in this formulation and were thus more interpretable. We replaced *QuestionnaireScore* in the equation described previously with the three psychiatric dimensions scores (*Anxious-depression*, *Compulsivity*, *Social withdrawal*; all z-scored) entered as predictors. The model was: ERN ~ *Anxious-depression* + *Compulsivity* + *Social withdrawal*.

### 2.14 Goal-directed learning

Participants also completed a reinforcement learning task (Daw et al., 2011) that enabled individual estimations of goal-directed (“model-based”) learning, which has previously been shown to be deficient in high compulsive individuals (Gillan et al., 2016). Briefly, the task consisted of two stages; in the first stage, participants had to choose between two items that had different probabilities of transitioning (rare: 30% or common: 70%) to one of two possible second stages. In the second stage, participants again had to choose between another two items which were associated with a distinct probability of being rewarded that drifted slowly over time. Individuals performing in a goal-directed (model-based) manner would make decisions based on the history of rewards and the transition structure of the task, as opposed to individuals who disregarded the transition structure and made decisions solely on the history of rewards (‘model-free’). To quantify goal-directed behaviour, we implemented a logistic regression model testing if participants’ choice behaviour was influenced by the reward, transition and their interaction of the previous trial. We then tested the relationship of psychiatric dimensions with goal-directed learning by including the three factors (*Anxious-depression*, *Compulsivity*, *Social withdrawal*) into the basic model as z-scored predictors. Note that inclusion of age and IQ in the model did not change the pattern of results. See Supplemental Methods for details of the regression equations.

The code and data to reproduce the ERN analyses of the paper are freely available at https://osf.io/vjda6/.

## 3 Results

Participants (N = 196) from a majority student sample completed an arrow-version of the Flanker task, a short IQ evaluation and a battery of self-report questionnaires assessing a range of psychiatric symptoms (see Methods). Individual item-level responses on these questionnaires were transformed into scores for three transdiagnostic dimensions using weights defined in a prior study (Gillan et al., 2016); anxious-depression, compulsive behaviours and intrusive thought and social withdrawal.

### 3.1 Behavioural results

Across participants, mean error rates ranged from 1.97% to 38.24% (mean (M) = 11.55%, standard deviation (SD) = 7.64%) and mean response times (RT) ranged from 123.73 ms to 472.55 ms (M =275.70 ms, SD = 67.59 ms). We observed basic behavioural patterns expected of the task. Mean error rates increased for incongruent trials (M = 15.58%, SD = 10.08%) relative to congruent trials (M = 3.61%, SD = 4.58%) (*t*_195_ = 19.97, 95% confidence interval (CI) [0.11, 0.13], *p* < 0.001). Mean RTs were shorter for congruent trials (M = 234.46 ms, SD = 66.51 ms) versus incongruent trials (M = 296.03 ms, SD = 70.16 ms) (*t*195 = −33.38, 95% CI [−0.07, −0.06], *p* < 0.001). Mean RTs were also shorter for error (mean =212.47 ms, SD =75.20 ms) as compared to correct (M = 283.23 ms, SD = 66.14 ms) trials (*t*_195_ = −22.41, 95% CI [− 0.08, −0.06], *p* < 0.001). Lastly, post-error mean RTs (M = 294.44 ms, SD = 88.47 ms) were slower than post-correct mean RT (M = 274.51 ms, SD = 68.38 ms) (*t*_195_ =6.13, 95% CI [0.01, 0.03], *p* <0.001). Error rate and RT distributions are visualised in Supplemental Fig. S1.

### 3.2 Response-locked event related potentials (ERPs)

**Fig. 1:**
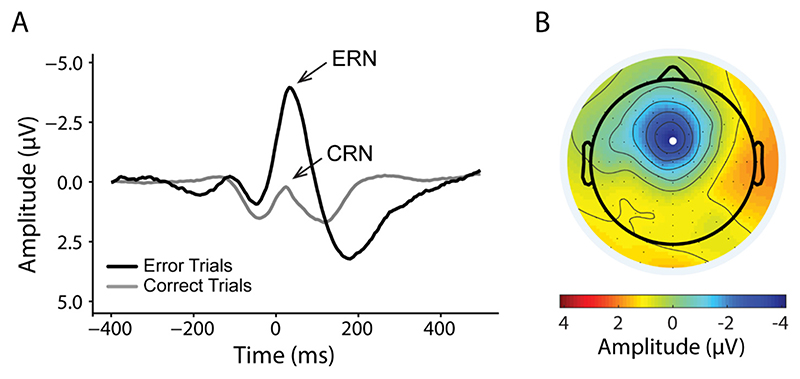
Error-related negativity (ERN). (A) Response-locked grand average waveforms for error and correct responses at electrode FCz. Negative values are plotted upwards. Event-related potential components are labelled: ERN: error-related negativity; CRN: correct-related negativity. (B) Scalp map displays the voltage distribution at 37.61 ms, the grand average latency of the most negative peak for error trials. Electrode FCz position is indicated with a white dot.

Grand-average ERP waveforms at electrode FCz are presented in Fig. 1A for the ERN and CRN. ERN waveforms contained an average of 51.88 (SD = 33.09) error trials per participant while the CRN waveform was constructed with average of 404 (SD = 58.78) correct trials. Across participants with the adaptive mean method, the ERN exhibited an amplitude of − 3.11 μV (SD = 2.79 μV) while the CRN had an amplitude of 0.30 μV (SD = 1.89 μV). Paired *t*-test indicated more pronounced negativities for the ERN than CRN (*t*_195_ = −16.66, 95% CI [−3.82, − 3.01], *p* <0.001) within-subject. Split half-reliability was high for both measures (ERN: *r* = 0.90; CRN: *r* = 0.98), confirming the suitability of this measure for between-subject analysis.

### 3.3 ERN, questionnaire scores and transdiagnostic dimensions

**Fig. 2:**
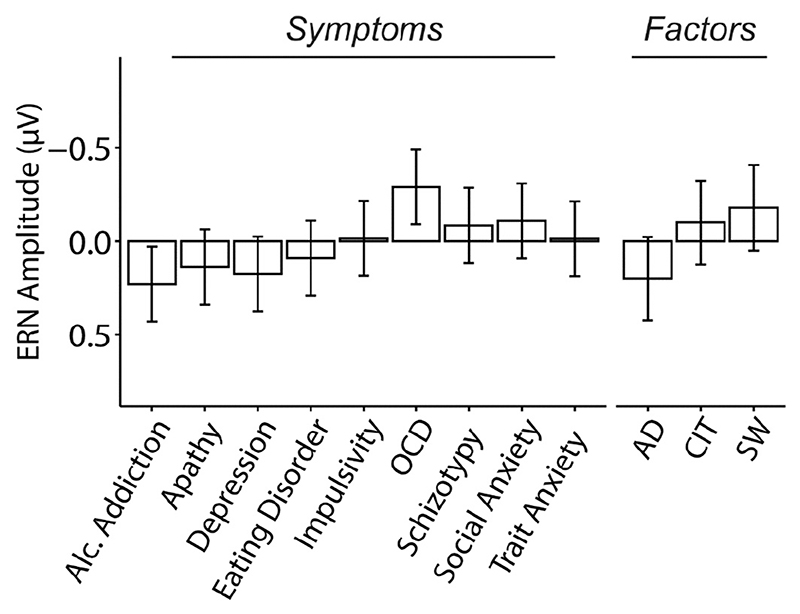
Non-significant associations between ERN amplitude and self-reported psychopathology. Associations between ERN amplitude with questionnaire total scores or transdiagnostic dimension scores (anxious-depression (AD), compulsive behaviour and intrusive thought (CIT) and social withdrawal (SW)). Error bars denote standard errors. Each questionnaire score was examined in a separate regression, whereas dimensions were included in the same model. The Y-axis indicates the change in ERN amplitude as a function of 1 standard deviation (SD) increase of questionnaire or dimension scores. See Table 1.

We tested if ERN amplitudes were associated to the self-reported questionnaire scores. In contrast to our hypothesis, none of the psychiatric questionnaires showed a significant relationship to ERN amplitude (all *p* > 0.15, where *p* < 0.005 is the Bonferroni corrected significance threshold) (Fig. 2 and Table 1). For the interested reader, we conducted unplanned, supplementary analyses to test for consistency of our findings across different methods of ERN quantification, electrode site and reaction times (speed-accuracy trade off (Arbel and Donchin, 2009; Gehring et al., 1993)). The patterns of results were remarkably similar: no symptom was significantly related to ERN amplitude in surrounding electrode sites (all *p* > 0.08, uncorrected) (Supplemental Fig. S6) or with three other ERN quantification methods (all *p* > 0.10, uncorrected) (Supplemental Fig. S7). Two other ERN measures that controlled for CRN variation (ΔERN and ERN_resid_) also did not reveal any significant associations (all *p* > 0.12, uncorrected) (Supplemental Fig. S8 and Table S3). Inclusion of error rate, demographics or medication status did not affect the pattern of results (all *p* > 0.09, uncorrected) (Supplemental Methods). Transdiagnostic phenotyping did not provide a better explanation for the data, with none of the transdiagnostic dimensions significantly associated to ERN amplitude (all *p* > 0.18, uncorrected) (Fig. 2 and Table 1).

### 3.4 Goal-directed control and compulsivity

**Fig. 3:**
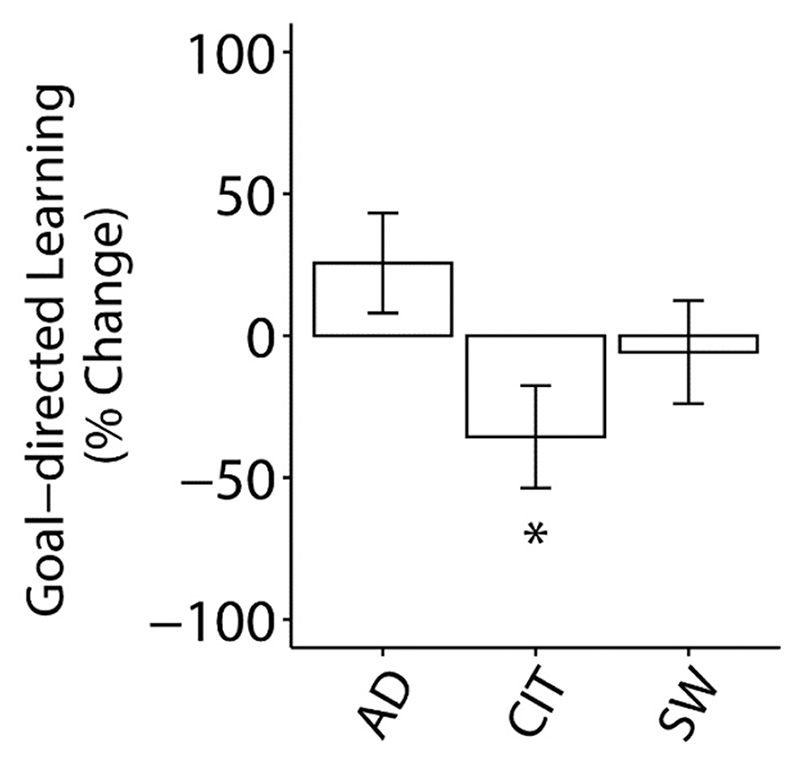
Associations between goal-directed learning and psychiatric dimensions (anxious-depression (AD), compulsive behaviour and intrusive thought (CIT) and social withdrawal (SW)) (N = 196). Error bars denote standard errors. Factors were included in the same model. The Y-axis indicates the percentage change in goal-directed learning as a function of 1 SD increase of dimension scores. *p < 0.05.

For comparison purposes, we also assessed goal-directed learning in the same sample using the two-step reinforcement learning task (Daw et al., 2011). Split half-reliability was *r* = 0.71 for this measure. In prior work, the compulsivity dimension was associated with reduced goal-directed learning (Gillan et al., 2016). We replicated this finding (*β* = −0.07, *SE* = 0.04, *p* < 0.05) (Fig. 3), suggesting that the dimension scores obtained from this general population sample were valid, providing a comparator for interpreting the effect size of ERN trends in the present work.

## 4 Discussion

In the present paper, we investigated if ERN abnormalities commonly observed in a range of psychiatric disorders could be explained by a transdiagnostic dimension characterised by high levels of anxious-depression. Fundamental to this was the replication of existing associations of ERN amplitude shifts with the clinical phenotypes such as OCD and trait anxiety, but to our surprise we could not detect any significant associations of any symptoms with the ERN. Reformulating questionnaires into transdiagnostic dimensions did not improve signal.

We considered several possible explanations for the data. First, that the range of psychopathology sampled was insufficiently high to detect associations with the ERN. We intentionally enriched our sample by including 8 patients from a local anxiety clinic (who were starting group therapy) to protect against this possibility. This was not necessary; the rest of the sample exhibited high rates of psychopathology (Supplemental Fig. S3), consistent with the documented characteristics of university students (Auerbach et al., 2018; Bayram and Bilgel, 2008; Evans et al., 2018). Excluding the 8 patients recruited from an anxiety disorder clinic, 37.97% of the sample who were assessed with a standard psychiatric interview (M.I.N.I., see Methods) presently met criteria for at least one disorder. In terms of the range of self-report symptoms, 25.51% (N = 50) scored ≥21 on the OCI-R and 54.59% (N = 107) scored >41 on the STAI, the standard clinical threshold for OCD and anxiety for the respective instruments (Ercan et al., 2015; Foa et al., 2002).

Second, we ensured that our data, both self-report and electrophysiological, were valid. Internal consistency measures were high for all questionnaires (Supplemental Table S1). We note that the transdiagnostic dimensions utilised here were defined from a prior study (Gillan et al., 2016) and not derived from our current data. The factor structure has been replicated in two other independent datasets (Rouault et al., 2018; Seow and Gillan, 2020) indicating it is reproducibile. But perhaps the strongest evidence for the validity of the transdiagnostic dimensions structure we employed are two replications with respect to the specific association between compulsive behaviour and intrusive thought scores and goal-directed planning; one observed in the present study and another via a separate research group (Patzelt et al., 2019). Speaking to the divergent validity of the factors, anxious-depression and social withdrawal are linked to different aspects of cognition. For example, dissociable metacognitive abnormalities have been observed for anxious-depression and compulsive dimensions (Rouault et al., 2018; Seow and Gillan, 2020), and excessive deliberation has been specifically linked to the social withdrawal dimension (Hunter et al., 2019).

Contextualising these data with the broader literature, it is possible that ERN abnormalities are more sensitive to the categorical comparison of patients versus controls than dimensional variation in the general population. Several OCD patient studies did not find any correlation with symptom severity and ERN amplitude within patient groups (Carrasco et al., 2013a; Endrass et al., 2008; Riesel, 2019; Riesel et al., 2017, 2014). The ERN remains elevated in OCD despite successful treatment (Hajcak, 2006; Ladouceur et al., 2018; Schrijvers et al., 2009) and elevations are also observed in unaffected first-degree relatives of patients (Carrasco et al., 2013a; Riesel et al., 2019). As such, the ERN has been couched a psychiatric *vulnerability endophenotype* (Riesel, 2019). Nonetheless, our individual differences approach should have been able to pick up these trait effects along the continuum of scores, regardless of the subtleties of state-based fluctuations.

In terms of the ERN itself, we were able to reproduce all the expected behavioural (Supplemental Figs. S1 and S2) and electrophysiological patterns this task was expected to elicit, suggesting that there were no issues with data quality. Our paradigm consisted of twice as many incompatible trials than compatible trials, which was intended to induce higher conflict frequency to increase the number of errors made and increase statistical power. As this ratio is not commonly employed, we cannot exclude the possibility that this may have affected our ability to detect associations between the ERN and our clinical measures. Prior work has suggested that task type (e.g. response-conflict versus probabilistic tasks) and task difficulty may alter ERN activity in OCD (Riesel, 2019). Specifically, for the Flanker task, increasing difficulty with shorter response times and poorer visual contrast have been observed to abolish ERN differences between groups of high/low OC symptoms (Kaczkurkin, 2013). Though this was different to the unique feature of our task design, the higher ratio of incompatible to compatible trials we used has been previously linked to reduced ERN amplitudes (Bartholow et al., 2005). Thus, it is possible that the ratio we employed may have dampened our ability to observe expected psychopathological effects with the ERN.

However, an alternative explanation for these data is that ERN associations with psychopathology are smaller than previously assumed. Notably, effects of OCD symptoms trended in the predicted direction, where individuals who scored higher on this questionnaire had a larger ERN. Likewise, the trend was for alcohol addiction to be associated with a blunted ERN, consistent with the previous literature. Recent reports add support to this conclusion. Two meta-analyses noted that overall effect sizes for anxiety or OCD traits for enhanced ERN were relatively small (Cavanagh and Shackman, 2015; Pasion and Barbosa, 2019) and another that assessed the effect size of ERN amplitude shifts in OCD (Riesel, 2019) noted that larger effect sizes were associated with smaller sample publications, suggesting publication bias. Indeed, when publication bias was accounted for in examining anxiety-ERN associations, a smaller effect size than earlier studies emerged (Saunders and Inzlicht, 2020). Our findings, based on a relatively large sample size (N = 196), were comparable to those in Saunders and Inzlicht (2020). Specifically, they found no significant overall relationship between anxiety and the ERN in a meta-analysis for *volunteer* (i.e. non-clinical) samples (Pearson’s *r* = − 0.06) once publication bias was accounted for. The anxiety-ERN effect size we report here is somewhat smaller again (*r* = −0.005), which may be explained by the influence of depression. Previous studies have observed that depressive symptoms can reduce ERN amplitudes in anxiety (Weinberg et al., 2016, 2015b, 2012a) and our dependent measure, the STAI, measures aspects of depression and indeed correlates highly with a separate instrument measuring depression (*r* = 0.75 in the present study). Saunders and Inzlicht (2020) examined a broader range of self-report measures, many of which were more directly targeted at worry and other anxious symptoms—which might have increased their effect size. Indeed, when we controlled for depressive symptoms in the present study, we observed that the association between anxiety and the ERN was larger with (standardised) *β* = −0.12 (see Supplement), though it remains non-significant.

It is perhaps important, nonetheless, that our transdiagnostic framework did not perform better in relative terms. Returning to our hypothesis, we found no evidence that anxious-depression might be responsible for the commonly observed enhancement of the ERN in anxiety disorders. In fact, the direction of this non-significant effect was in the opposite direction. This finding might indicate that the dimensional framework we applied is not apt to capture variation in the ERN, which prior work has suggested has a partially dissociable relationship to depression and anxiety (Weinberg et al., 2016, 2015b, 2012a). As the transdiagnostic dimensions utilised in this study have previously shown specific associations with other cognitive deficits such as goal-directed planning (Gillan et al., 2016; Seow et al., 2020), metacognition (Rouault et al., 2018; Seow and Gillan, 2020) and aspects of socially-framed deliberation (Hunter et al., 2019), rather than speaking to the validity of this structure itself, this could suggest that the ERN operates at a different level of the psychiatric hierarchy to these processes—perhaps one that dissociates anxiety and depression more effectively.

The transdiagnostic dimensions utilised in this study were not intended to be fixed and final. Future research should explore alternatives to the framework employed here to investigate if a dimensional structure exists that can explain the common ERN patterns seen across psychiatric disorders. For instance, the internalising-externalising spectrum (Krueger, 1999; Krueger et al., 1998) is a model that has previously been discussed in relation to disambiguating ERN effects across psychiatry. The internalising dimension (encapsulating disorders of negative emotionality like depression and anxiety) has been linked to enhanced ERN amplitudes while the externalising dimension (comprising of disorders with aggression and impulsion like drug and substance use disorders) with reduced ERN amplitudes (Olvet and Hajcak, 2008; Pasion and Barbosa, 2019). These dimensions are similar to those used in this study in some ways; for instance, the internalising dimension would include anxious-depression. However, OCD is often considered as an internalising disorder, while here we considered compulsivity as distinct dimension from anxious-depression. While some groups have tested the internalising-externalising ERN hypothesis, the investigations have mainly been in paediatric samples (Kessel et al., 2016; Meyer and Klein, 2018; Troller-Renfree et al., 2016), and importantly, internalising and externalising phenotypes are often subjectively defined across studies. While promising, future studies intending to investigate the potential of alternative dimensional hypotheses of the ERN should ensure the validity and replicability of these frameworks.

## 5 Conclusions

In recent years, several authors have highlighted the potential for a transdiagnostic framework at reconciling the broad range of ERN patterns in the literature (Gillan et al., 2017; Pasion and Barbosa, 2019; Riesel, 2019). The present paper is timely, being the first study to apply an expansive and empirically robust transdiagnostic approach that directly addresses the issue of co-occurring symptoms in a large sample. To our surprise, despite being relatively well-powered, we were unable to replicate previously observed associations with various aspects of mental health. The application of a transdiagnostic methodology did nothing to remedy that. Future research in this area might take note that even larger samples than previously assumed are likely needed to delineate robust associations between self-report mental health and the ERN in general population samples.

## Supplementary Material

Supplementary data to this article can be found online at https://doi.org/10.1016/j.ijpsycho.2020.09.019.

Supplementary info

## Figures and Tables

**Table 1:** Associations between ERN amplitude and total scores of self-report psychiatric questionnaires or transdiagnostic dimensions. SE = standard error. For psychiatric questionnaires, each row reflects the (uncorrected for multiple comparisons) results from an independent analysis where each psychiatric questionnaire score was regressed against ERN amplitude. For transdiagnostic dimensions, all three dimensions scores were included in the same regression model.

Psychiatric questionnaire	*β* (*SE*)	*z*-Value	*p*-Value
Alcohol addiction	0.23 (0.20)	1.14	0.25
Apathy	0.14 (0.20)	0.68	0.50
Depression	0.18 (0.20)	0.88	0.38
Eating disorder	0.09 (0.20)	0.45	0.65
Impulsivity	−0.05 (0.20)	−0.08	0.94
OCD	−0.29 (0.20)	−1.45	0.15
Schizotypy	−0.08 (0.20)	−0.42	0.67
Social anxiety	−0.11 (0.20)	−0.54	0.59
Trait anxiety	−0.01 (0.20)	−0.07	0.95
Transdiagnostic dimension	*β* (*SE*)	*t*-Value	*p*-Value
Anxious-depression	0.29 (0.22)	1.34	0.18
Compulsive behaviour and intrusive thought	−0.03 (0.22)	−0.14	0.86
Social withdrawal	−0.20 (0.22)	−0.91	0.36
