# Supplementary material for: A dimensional investigation of error-related negativity (ERN) and self-reported psychiatric symptoms
Source: Int J Psychophysiol. Author manuscript; Available in PMC 2021 Dec 23. (PMC7612131; doi:10.1016/j.ijpsycho.2020.09.019)
Supplement: Supplementary info [file EMS140561-supplement-Supplementary_info.pdf]

Supplemental Information

for

A dimensional investigation of error-related negativity (ERN) and self-reported psychiatric symptoms

Supplemental Methods

ERN, demographics and error rate. In existing work, age, gender and IQ (Falkenstein et al., 2001; Fischer et al., 2016; Larson et al., 2016; Zijlmans et al., 2019) yield various relationships with ERN amplitude. We explored their effects on ERN in our data. IQ (β = -0.38, *SE* = 0.20, *p* = 0.06) and age (β = 0.39, *SE* = 0.20, *p* = 0.05) showed a trending effect with ERN amplitude shifts, while gender not associated (both $p < 1$). We also observed that error rate was related to the ERN (β = 0.40, *SE* = 0.20, *p* = 0.04). However, inclusion of age and IQ nor error rate did not change the effect of questionnaires scores on ERN amplitude (all $p > 0.13$, uncorrected).

ERN and medication status. The ERN has also been previously influenced by various psychotropic medication (Bates et al., 2002; de Bruijn et al., 2006; Endrass et al., 2008; Henderson et al., 2006; Riba et al., 2005). Only 31 (15.82%) participants were currently medicated for a mental health issue, which was too small a sample to conduct analyses divided by medication type. Nonetheless, we investigated if medication status was related to ERN amplitude. It was not (β = 0.80, *SE* = 0.55, *p* = 0.15), and neither did inclusion of medication status significantly modulate the effect of questionnaires scores on ERN amplitude (all $p > 0.09$, uncorrected).

ERN amplitude measures. In the literature, there are various ways to quantify ERN amplitude (Clayson et al., 2013). Here we report the supplementary analyses

showing that the main results were not due to our chosen analysis approach—whether it was from electrode site (Supplemental Figure S6) or ERN quantification method (Supplemental Figure S7). For non-adaptive mean, ERN amplitude calculated as the mean of ±40ms at 37.61ms post-response, which was the mean latency of the most negative peak across participants. For peak, the most negative peak was identified, and amplitude was extracted, for each participant by searching for the largest preceding negativity within -20ms to 120ms post-response. For trough-peak, the trough was identified for each participant by searching for the largest preceding positivity within -100ms before the peak. The amplitude of this positive peak was then subtracted from the negative peak amplitude.

ERN controlled for CRN variation. A common method thought to isolate activity specific to error monitoring is calculated by the subtraction of the CRN from the ERN i.e. ERN-CRN (ΔERN) (Gehring et al., 1993). However, using the subtraction method is conceptually problematic as the ERN and CRN are highly correlated across individuals (here, ERN and CRN correlate: $r = 0.30$, $p < 0.001$). This is because difference scores are not independent from the constituent measures (i.e. not an error processing measure independent of the CRN) and may conflate effects relating to either signal (Meyer et al., 2017). An alternative approach to control for variation of the CRN is to use the variation left over from a regression of CRN predicting ERN (ERN_{resid}) as the ERN amplitude measure. ERN_{resid} was correlated to ERN ($r = 0.95$, $p < 0.001$) but not to the CRN ($r = \sim 0$, $p = 1$), suggesting that it specifically indexes error-related activity and is a more interpretable measure. We report the associations of these two different ERN measures, ΔERN and ERN_{resid} with questionnaire scores

(Figure S8 and Table S3), and note both findings do not reveal any significant effects (all $p > 0.12$, uncorrected).

ERN, depression and anxiety. Previous studies have suggested that depression can reduce the increased ERN amplitudes effect associated with anxiety (Weinberg et al., 2016, 2015, 2012). We tested if this was true in our data by regressing depression and anxiety total scores against ERN estimates in the same model. Both effects remained non-significant; but the direction of effects was perhaps more representative of the literature with anxiety leaning towards a larger ERN ($\beta = -0.33$, $SE = 0.30$, $p = 0.27$; standardised $\beta = -0.12$ (a metric that is comparable to r (Peterson and Brown, 2005)) and depression towards a smaller ERN ($\beta = 0.43$, $SE = 0.30$, $p = 0.16$).

Goal-directed learning. The same sample of participants (N = 234) completed the two-step reinforcement learning task (Daw et al., 2011). Several exclusion criteria were applied to ensure data quality, on a rolling basis. i) Participants who responded with the same key in stage one >90% (n = 135) of the time (N = 10). ii) Participants whose probability of staying after common, rewarded trials was less than 5% likely to be at chance, based on a binomial distribution with 50% (chance) probability and the total number of common-rewarded trials experienced by each participant (N = 11). iii) Participants who missed >20% (n = 30) of the trials were excluded (N = 3). (iv) Participants who incorrectly responded to a “catch” question within the questionnaires: “If you are paying attention to these questions, please select ‘A little’ as your answer” were excluded (N = 7). (v) As we intend to analyse the EEG data collected for this task, we additionally excluded participants whose EEG data were

incomplete (N = 5) or corrupt (N = 2) from the analysis. 38 participants (16.24%) were excluded in total, leaving 196 participants for analysis. To clean the task data, we excluded individual trials with very fast reaction times (<150ms) reflecting inattention or poor responding. Including missed trials, a total of 1114 (3.77%) trials were excluded.

To estimate goal-directed learning, we performed logistic regression via mixed-effects models with the *lme4* package in R, with Bound Optimization by Quadratic Approximation (bobyqa) with 1e5 functional evaluations. The basic model tested if participants' choice behaviour to *Stay* or switch relative to previous choice (stay: 1, switch: 0) was influenced by the previous trial's *Reward* (rewarded: 1, unrewarded: -1), *Transition* (common (70%): 1, rare (30%): -1) and their interaction, with age, gender and IQ as z-scored fixed-effects covariates. Within-subject factors (the intercept, main effects of reward, transition, and their interaction) were taken as random effects (i.e. allowed to vary across participants). In syntax of R, the model was: Stay ~ Reward * Transition + (Reward * Transition + 1 | Subject). The interaction effect between Reward and Transition was significant, indicating a contribution of goal-directed learning to choice behaviour ($\beta = 0.20$, $SE = 0.03$, $p < 0.001$). To test if symptom dimensions were associated with goal-directed learning deficits, we included the total scores of the three dimensions (anxious-depression, compulsive behaviour and intrusive thought ('compulsivity'), social withdrawal) as z-scored fixed effect predictors into the basic model described above. The model was: Stay ~ Reward * Transition + (*Anxious-depression* + *Compulsivity* + *Social withdrawal*) + (Reward * Transition + 1 | Subject). The extent to which a dimension is related to deficits in goal-directed learning was indicated by the presence of a

significant Reward*Transition**Dimension* interaction. In prior work, age and IQ were associated to model-based planning (Gillan et al., 2016). Inclusion of these demographics did not change the pattern of effect to compulsivity (β = -0.08, SE = 0.04, p = 0.04).

Supplemental Figures and Tables

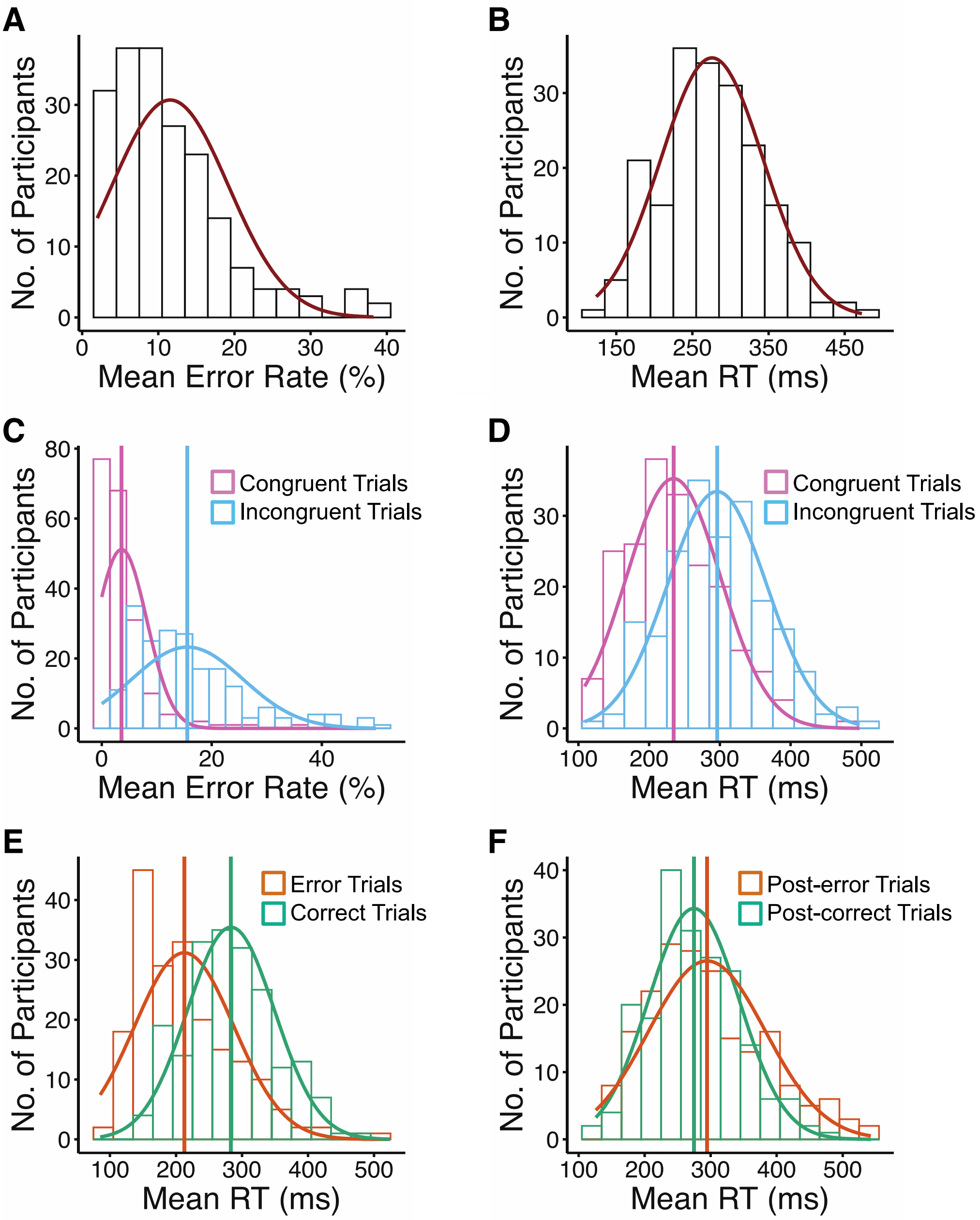

Figure S1. *Across participants, the distribution of:*

(A) *Mean error rate.*

(B) *Mean response time (RT).*

(C) *Mean RT by trial congruency.*

(D) *Mean RT by trial congruency.*

(E) *Mean RT by trial accuracy.*

(F) *Mean RT by post-trial accuracy.*

Vertical lines denote mean error rate/RT for respective trial type.

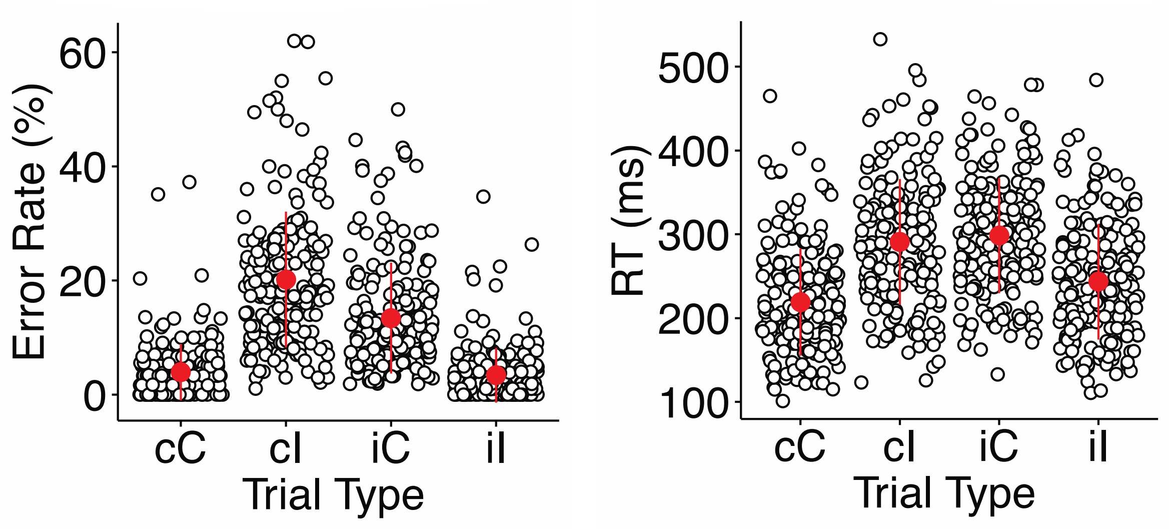

Figure S2. *Mean error rate and response times (RT) for various trial types. cC: congruent trials preceded by a congruent trial, cI: incongruent trial preceded by a congruent trial, iC: congruent trial proceeded by an incongruent trial), iL: incongruent trial preceded by an incongruent trial). White dots represent individual participants, red marker indicates mean and SD.*

Conflict adaptation. Conflict adaptation effects refer to the phenomenon wherein previous-trial congruency affects current-trial performance, which have consistently been shown as behavioural adjustment in error rates and RTs in Flanker tasks (Clayson and Larson, 2011; Larson et al., 2016). We replicate these effects, where mean error rates were smaller for iI than for cI trials (t_{195} = -22.08, 95% CI [-0.18, -0.15], p < 0.001) and for cC relative to iC trials (t_{195} = -15.76, 95% CI [-0.11, -0.08], p < 0.001). Additionally, mean RTs were shorter for iI compared to cI (t_{195} = -24.24, 95% CI (Confidence Interval) [-0.05, -0.04], p < 0.001) and for cC relative to iC (t_{195} = -32.48, 95% CI [-0.08, -0.07], p < 0.001) trials.

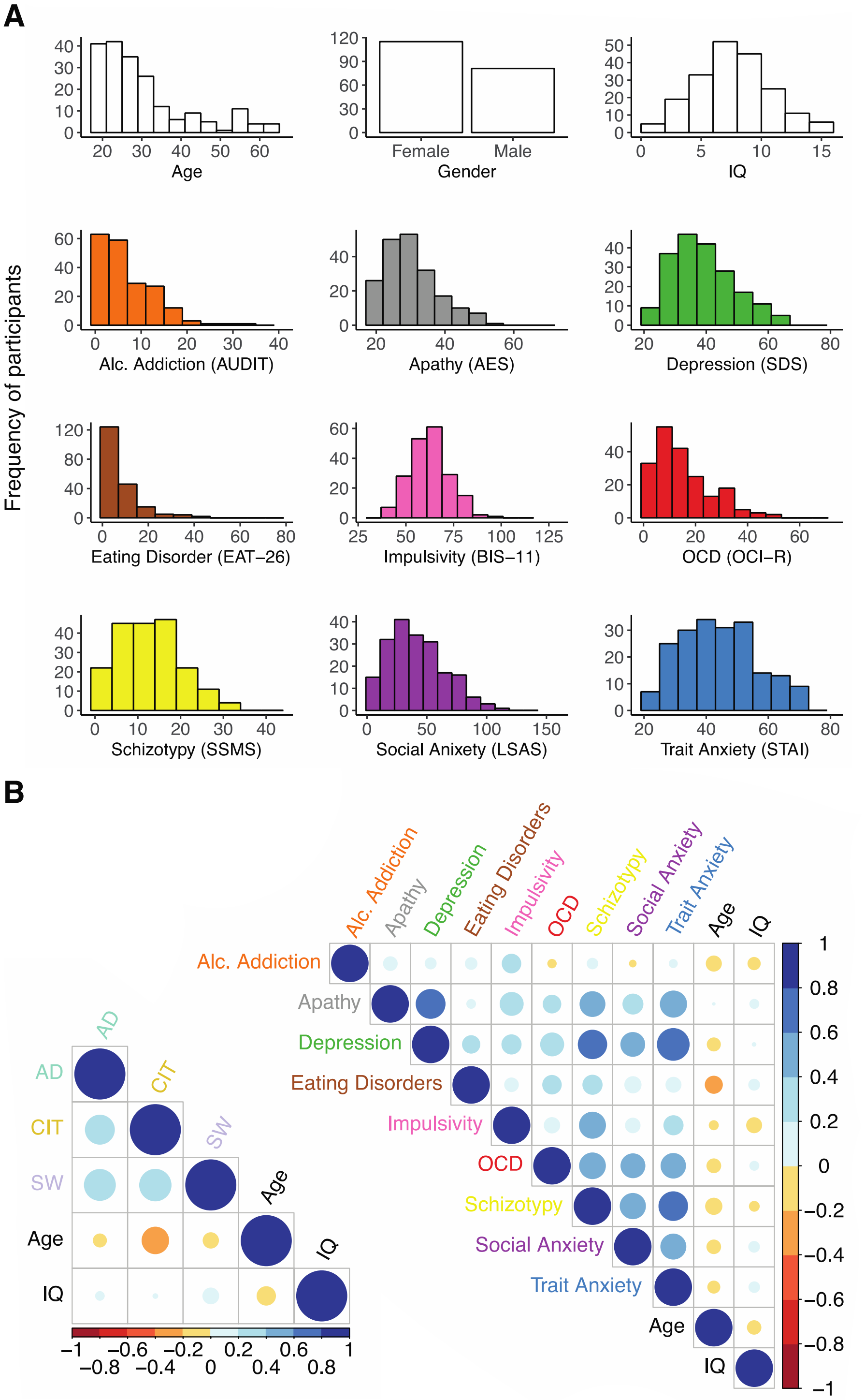

Figure S3. *Demographics and self-reported psychopathology spread.*

(A) *Age, IQ and psychiatric symptoms score distributions across participants.*

(B) *Correlation matrix of mean scores of the nine pyschiatric questionnaires or transformed dimension scores (AD: anxious-depression, CIT: compulsive behaviour and intrusive thought, SW: social withdrawal), including age and IQ. Colour scale indicates correlation coefficient, size of colour patch indicates significance.*

Table S1. *Pearson's correlations between total questionnaire scores. Cronbach's Alpha was used to calculate reliability for questionnaires.*

	Alcohol Addiction	Apathy	Depression	Eating Disorder	Impulsivity	OCD	Schizotypy	Social Anxiety	Trait Anxiety
Alcohol Addiction									
Apathy	0.14								
Depression	0.09	0.62							
Eating Disorder	0.10	0.06	0.22						
Impulsivity	0.26	0.39	0.27	0.14					
OCD	-0.05	0.23	0.39	0.25	0.18				
Schizotypy	0.08	0.46	0.60	0.27	0.51	0.49			
Social Anxiety	-0.03	0.30	0.42	0.20	0.16	0.43	0.48		
Trait Anxiety	0.05	0.51	0.75	0.16	0.27	0.45	0.61	0.46	
Reliability	0.87	0.86	0.85	0.86	0.81	0.89	0.85	0.92	0.93

Table S2. *Pearson's correlations between transdiagnostic dimensions scores (AD: 'anxious-depression', CIT: 'compulsive behaviour and intrusive thought', SW: 'social withdrawal').*

	AD	CIT	SW
AD			
CIT	0.33		
SW	0.38	0.39	

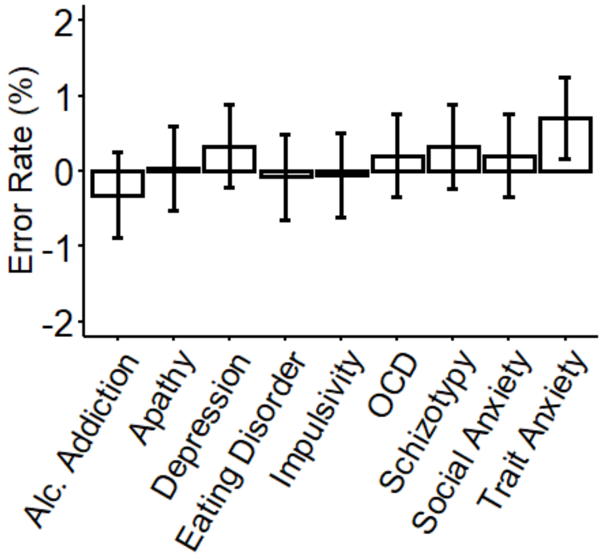

Figure S4. *Associations between questionnaire scores with mean error rate (%). Error bars denote standard errors. The Y-axes indicate the change in error rate as a function of 1 standard deviation (SD) increase of questionnaire scores. No questionnaire score was significantly associated to changes in error rate.*

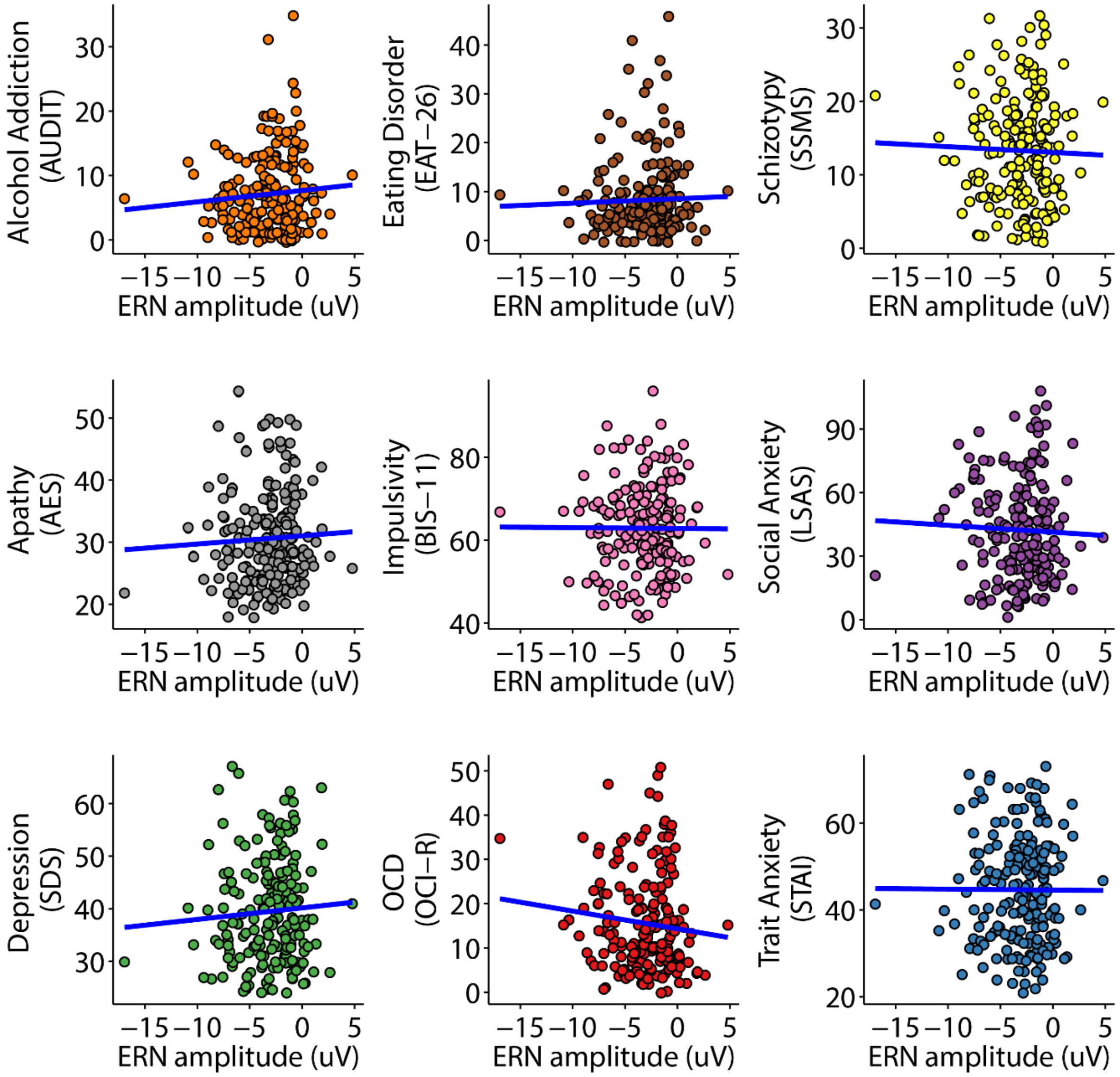

Figure S5. *Scatterplots of ERN amplitude and total questionnaire scores. Coloured markers represent an individual's total score for the corresponding questionnaire. See Figure 2 and Table 1. We note that a possible outlier appears to exist (ERN amplitude > -15 uV), but when the data point is removed, the associations between questionnaire scores and the ERN do not statistically differ from the original correlations (William's test of correlation difference: all z < 0.40 , all p > 0.69).*

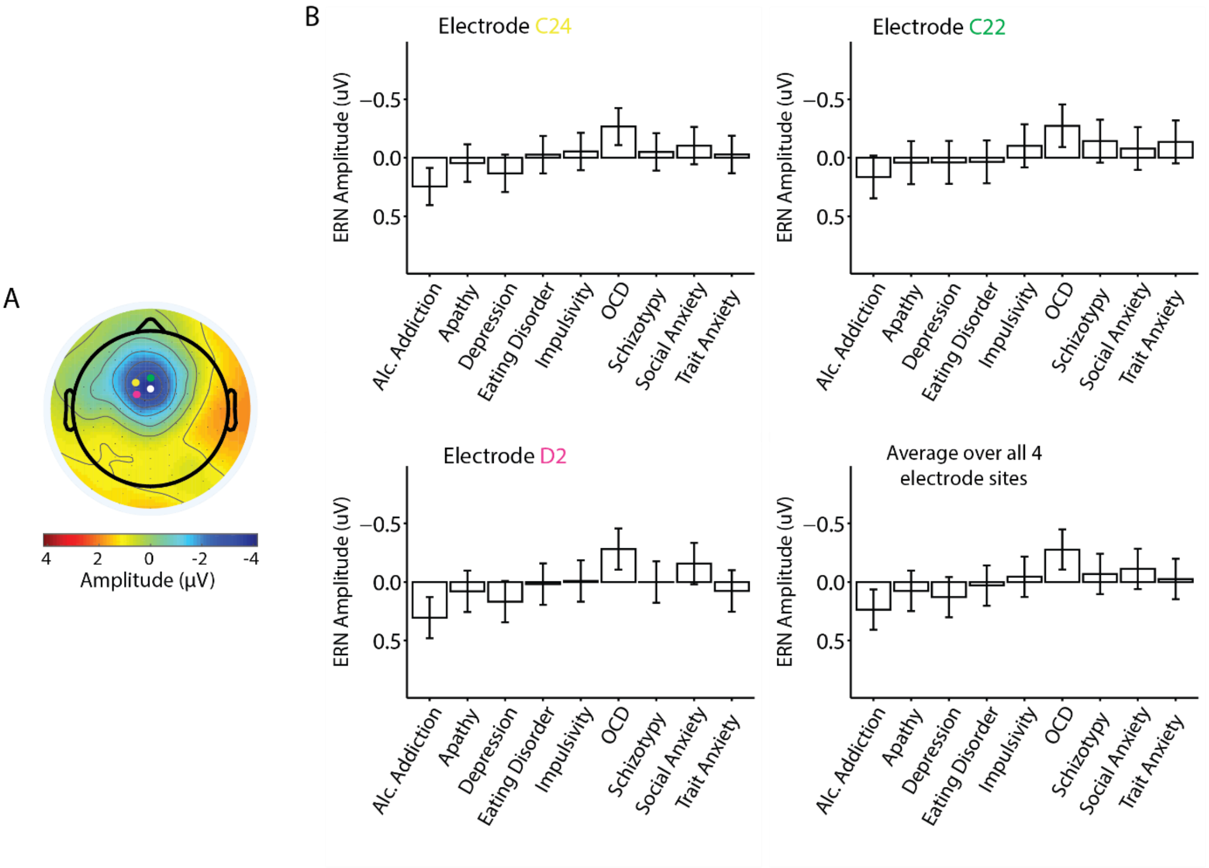

Figure S6. *ERN quantification at various electrode sites with the adaptive mean method.*

(A) *Scalp map displays the voltage distribution at 37.61ms, the average latency of the most negative peak. Coloured dots indicate electrode positions around ERN peak; FCz: white, C22: green, C24: yellow, D2: pink.*

(B) *Associations between questionnaire total scores with ERN amplitude quantified at various electrode sites. Error bars denote standard errors. The Y-axes indicates the change in in ERN amplitude as a function of 1 SD increase of questionnaire scores.*

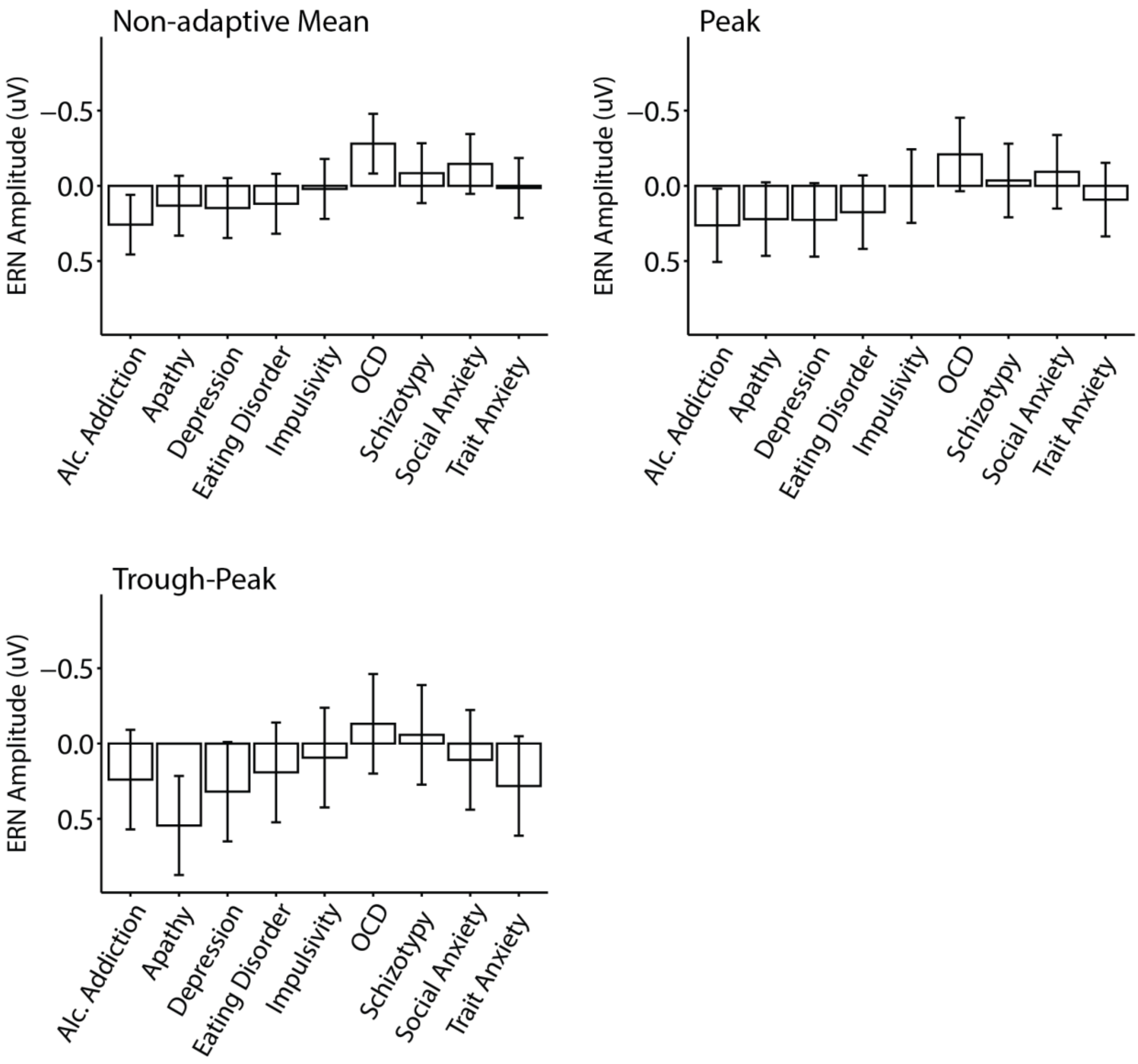

Figure S7. *Associations between psychiatric symptoms with ERN amplitude quantified by various methods at electrode C23 (FCz). Error bars denote standard errors. The Y-axes indicate the change in ERN amplitude as a function of 1 SD increase of questionnaire scores.*

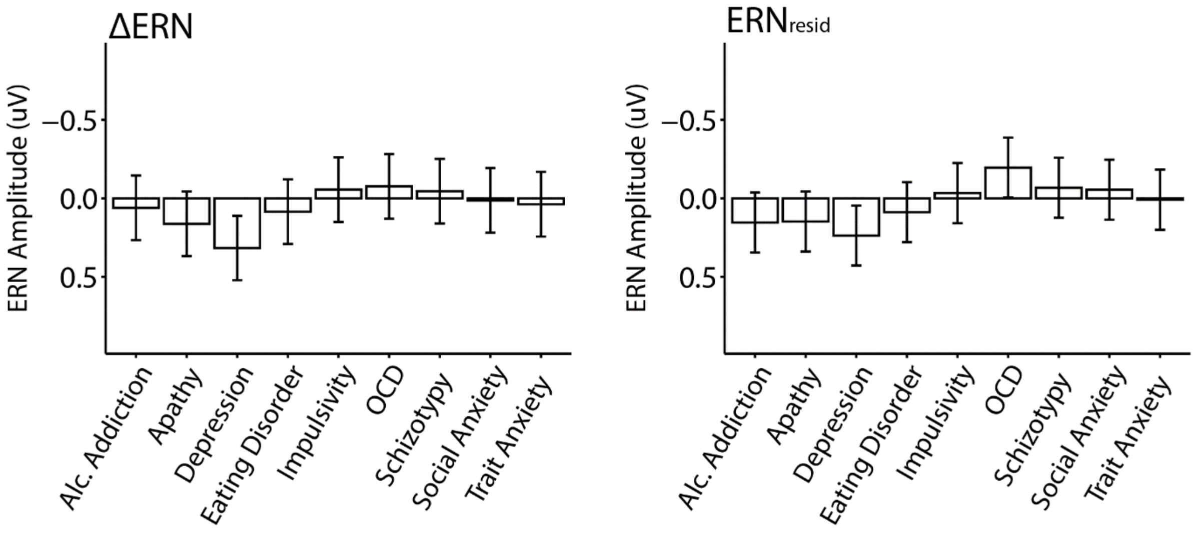

Figure S8. *Associations between psychiatric symptoms with ΔERN and ERN_{resid} amplitude measured at electrode C23 (FCz). Error bars denote standard errors. The Y-axes indicate the change in ERN amplitude as a function of 1 SD increase of questionnaire scores. See Table S3.*

Table S3: *Associations between ΔERN or ERN_{resid} amplitude with total scores of self-report psychiatric questionnaires or transdiagnostic dimensions. SE = standard error. For psychiatric questionnaires, each row reflects the (uncorrected for multiple comparisons) results from an independent analysis where each psychiatric questionnaire score was regressed against ERN amplitude. For transdiagnostic dimensions, all three dimensions scores were included in the same regression model. See Figure S8.*

	ΔERN			***ERN_{resid}***		
Psychiatric Questionnaire	*β (SE)*	*z-value*	*p-value*	*β (SE)*	*z-value*	*p-value*
Alcohol Addiction	0.06 (0.20)	0.29	0.77	0.15 (0.19)	0.81	0.42
Apathy	0.16 (0.20)	0.79	0.43	0.15 (0.19)	0.77	0.44
Depression	0.32 (0.20)	1.55	0.12	0.24 (0.19)	1.24	0.21
Eating Disorder	0.09 (0.20)	0.42	0.68	0.09 (0.19)	0.46	0.64
Impulsivity	-0.06 (0.20)	-0.27	0.79	-0.03 (0.19)	-0.17	0.86
OCD	-0.08 (0.20)	-0.37	0.71	-0.20 (0.19)	-1.02	0.31
Schizotypy	-0.05 (0.20)	-0.22	0.83	-0.07 (0.19)	-0.35	0.73
Social Anxiety	0.01 (0.20)	0.07	0.95	-0.05 (0.19)	-0.29	0.77
Trait Anxiety	0.04 (0.20)	0.18	0.86	0.009 (0.19)	0.04	0.96
Transdiagnostic Dimension	*β (SE)*	*t-value*	*p-value*	*β (SE)*	*t-value*	*p-value*
Anxious-depression	0.11 (0.23)	0.49	0.62	0.16 (0.21)	0.76	0.45
Compulsive behaviour and intrusive thought	-0.01 (0.22)	-0.06	0.95	-0.06 (0.21)	-0.29	0.77
Social withdrawal	-0.02 (0.24)	-0.10	0.92	-0.11 (0.22)	-0.51	0.61

Table S4: *Mini International Neuropsychiatric interview (M.I.N.I.) diagnostic information summary for participants who presently met the criteria for at least one DSM-V disorder (N = 38).*

Disorder	***Diagnosis***
Mood disorders	
Major depressive disorder	*18*
Suicide behavior disorder	*1*
Bipolar Disorder	*1*
Anxiety disorders	
Panic Disorder	*12*
Agoraphobia	*4*
Generalised Anxiety Disorder	*15*
Social Anxiety Disorder	*11*
Obsessive-Compulsive Disorder	*5*
Posttraumatic Stress Disorder	*0*
Substance use disorders	
Alcohol Use Disorder	*7*
Substance Use Disorder (Non-alcohol)	*9*
Psychotic Disorders	
Psychotic Disorders	*0*
Eating Disorders	
Anorexia Nervosa	*0*
Bulimia Nervosa	*1*
Binge-Eating Disorder	*4*
Other disorders	
Antisocial Personality Disorder	*1*

References

Bates, A.T., Kiehl, K.A., Laurens, K.R., Liddle, P.F., 2002. Error-related negativity and correct response negativity in schizophrenia. Clin. Neurophysiol. 113, 1454–1463. https://doi.org/10.1016/S1388-2457(02)00154-2

Clayson, P.E., Baldwin, S.A., Larson, M.J., 2013. How does noise affect amplitude and latency measurement of event-related potentials (ERPs)? A methodological critique and simulation study. Psychophysiology 50, 174–186. https://doi.org/10.1111/psyp.12001

Clayson, P.E., Larson, M.J., 2011. Conflict adaptation and sequential trial effects: Support for the conflict monitoring theory. Neuropsychologia 49, 1953–1961. https://doi.org/10.1016/j.neuropsychologia.2011.03.023

Daw, N.D., Gershman, S.J., Seymour, B., Dayan, P., Dolan, R.J., 2011. Model-based influences on humans' choices and striatal prediction errors. Neuron 69, 1204–1215. https://doi.org/10.1016/j.neuron.2011.02.027

de Bruijn, E.R.A., Sabbe, B.G.C., Hulstijn, W., Ruigt, G.S.F., Verkes, R.J., 2006. Effects of antipsychotic and antidepressant drugs on action monitoring in healthy volunteers. Brain Res. 1105, 122–129. https://doi.org/10.1016/j.brainres.2006.01.006

Endrass, T., Klawohn, J., Schuster, F., Kathmann, N., 2008. Overactive performance monitoring in obsessive-compulsive disorder: ERP evidence from correct and erroneous reactions. Neuropsychologia 46, 1877–1887. https://doi.org/10.1016/j.neuropsychologia.2007.12.001

Falkenstein, M., Hoormann, J., Hohnsbein, J., 2001. Changes of error-related ERPs with age. Exp. Brain Res. 138, 258–262. https://doi.org/10.1007/s002210100712

Fischer, A.G., Danielmeier, C., Villringer, A., Klein, T.A., Ullsperger, M., 2016. Gender Influences on Brain Responses to Errors and Post-Error Adjustments. Sci. Rep. 6, 24435. https://doi.org/10.1038/srep24435

Gehring, W.J., Goss, B., Coles, M.G.H., Meyer, D.E., Donchin, E., 1993. A Neural System for Error Detection and Compensation. Psychol. Sci. 4, 385–390. https://doi.org/10.1111/j.1467-9280.1993.tb00586.x

Gillan, C.M., Kosinski, M., Whelan, R., Phelps, E.A., Daw, N.D., 2016. Characterizing a psychiatric symptom dimension related to deficits in goal-directed control. Elife 5, e11305. https://doi.org/10.7554/eLife.11305

Henderson, H., Schwartz, C., Mundy, P., Burnette, C., Sutton, S., Zahka, N., Pradella, A., 2006. Response monitoring, the error-related negativity, and differences in social behavior in autism. Brain Cogn. 61, 96–109. https://doi.org/10.1016/j.bandc.2005.12.009

Larson, M.J., Clayson, P.E., Keith, C.M., Hunt, I.J., Hedges, D.W., Nielsen, B.L., Call, V.R.A., 2016. Cognitive control adjustments in healthy older and younger

adults: Conflict adaptation, the error-related negativity (ERN), and evidence of generalized decline with age. Biol. Psychol. 115, 50–63. https://doi.org/10.1016/j.biopsycho.2016.01.008

Meyer, A., Lerner, M.D., De Los Reyes, A., Laird, R.D., Hajcak, G., 2017. Considering ERP difference scores as individual difference measures: Issues with subtraction and alternative approaches. Psychophysiology 54, 114–122. https://doi.org/10.1111/psyp.12664

Peterson, R.A., Brown, S.P., 2005. On the use of beta coefficients in meta-analysis. J. Appl. Psychol. 90, 175. https://doi.org/10.1037/0021-9010.90.1.175

Riba, J., Rodríguez-Fornells, A., Münte, T.F., Barbanoj, M.J., 2005. A neurophysiological study of the detrimental effects of alprazolam on human action monitoring. Cogn. Brain Res. 25, 554–565. https://doi.org/10.1016/j.cogbrainres.2005.08.009

Weinberg, A., Klein, D.N., Hajcak, G., 2012. Increased error-related brain activity distinguishes generalized anxiety disorder with and without comorbid major depressive disorder. J. Abnorm. Psychol. 121, 885. https://doi.org/10.1037/a0028270

Weinberg, A., Kotov, R., Proudfit, G.H., 2015. Neural indicators of error processing in generalized anxiety disorder, obsessive-compulsive disorder, and major depressive disorder. J. Abnorm. Psychol. 124, 172. https://doi.org/10.1037/abn0000019

Weinberg, A., Meyer, A., Hale-Rude, E., Perlman, G., Kotov, R., Klein, D.N., Hajcak, G., 2016. Error-related negativity (ERN) and sustained threat: Conceptual framework and empirical evaluation in an adolescent sample. Psychophysiology 53, 372–385. https://doi.org/10.1111/psyp.12538

Zijlmans, J., Bevaart, F., van Duin, L., Luijks, M.J.A., Popma, A., Marhe, R., 2019. Error-related brain activity in relation to psychopathic traits in multi-problem young adults: An ERP study. Biol. Psychol. 144, 46–53. https://doi.org/10.1016/j.biopsycho.2019.03.014
